# Effect of home biofeedback treatment in patients with fecal incontinence: a pilot study

**DOI:** 10.1007/s10151-026-03384-8

**Published:** 2026-06-24

**Authors:** Gülşah Filiz Karpuz, Şakir Karpuz, Wafi Attaallah

**Affiliations:** 1 Department of General Surgery, Marmara University Pendik Training and Research Hospital, Fevzi Çakmak Mahallesi, Muhsin Yazıcıoğlu Caddesi No:10 Pendik, 34899 Istanbul, Turkey; 2 Department of General Surgery, Marmara University School of Medicine, Istanbul, Turkey; 3 Department of General Surgery, Anadolu Health Center, Kocaeli, Turkey

**Keywords:** Fecal incontinence, Biofeedback, Quality of life, Wexner score

## Abstract

**Background:**

Fecal incontinence is a common condition that markedly impairs quality of life. Anal biofeedback therapy is recommended as first-line treatment; however, its widespread use is limited by the need for repeated sessions, trained staff, high cost, and patient embarrassment, often resulting in poor adherence. This study aimed to evaluate the effectiveness of self-administered home-based biofeedback therapy and to determine factors associated with treatment success.

**Methods:**

This prospective, single-center study included adult patients presenting with fecal incontinence. Disease severity and quality of life were assessed using the Wexner score, fecal incontinence severity index (FISI), and fecal incontinence quality of life (FIQOL) scale. Baseline evaluations included anal manometry, electromyography, perianal magnetic resonance imaging, or endoanal ultrasonography. All patients underwent a 3-month home-based anal biofeedback program. Primary outcomes were changes in Wexner, FISI, and FIQOL scores after treatment.

**Results:**

Between September 2019 and January 2021, 24 patients were enrolled. The median Wexner score decreased significantly from 18 (4–20) to 6 (0–16) following therapy (*p* < 0.001). Similarly, the median FISI score improved from 48 (20–57) to 15 (0–45) (*p* < 0.001). Anal manometry demonstrated a significant increase in median maximum squeeze pressure from 20 (0–80) mmHg to 60 (17–96) mmHg (*p* < 0.001). Significant improvements were observed across all four FIQOL domains: lifestyle/coping, self-perception/sexuality, depression/anxiety, and embarrassment. Overall, 86% of patients showed improvement in Wexner scores, ranging from 11% to 100%, and 53% achieved a reduction greater than 50%.

**Conclusions:**

Self-administered home-based biofeedback therapy is an effective and practical treatment for fecal incontinence. Given its simplicity, accessibility, and cost-effectiveness, it represents a valuable alternative to conventional biofeedback therapy.

## Introduction

Fecal incontinence (FI) is a common condition that has a profound negative impact on patients’ quality of life [1]. According to the Rome IV criteria, FI is defined as the uncontrolled passage of fecal material occurring at least two to four times over a 4-week period within the past 6 months [2]. Despite its prevalence, fecal incontinence remains underrecognized by both the general public and healthcare providers. It may lead to several medical complications such as perianal dermatitis, urinary tract infections, and pressure ulcers, all of which contribute to a considerable economic and psychological burden [3].

The reported prevalence of FI ranges from 2.2% to 15%, though the actual rate is likely higher [4]. Prevalence varies with age and sex, being approximately 2–4% in the general population, 11% in men over 50 years, and 26% in women over 50 years of age. In primary care settings, FI prevalence has been reported as 13.4%, whereas in gastroenterology clinics it reaches up to 26% [5, 6]. Among nursing home residents, the prevalence may rise to 40% [7]. Complete continence depends on adequate pelvic floor function, intact anal sphincters, and normal rectal compliance and neurological control [8, 9].

The most common cause of FI is obstetric trauma. Clinically evident anal tears occur in approximately 3.3% of women after vaginal delivery [10], whereas endoanal ultrasonography reveals sphincter defects in up to 35% after the first vaginal delivery [11]. One-third of patients with sphincter damage develop symptoms of incontinence [4]. The second most frequent cause is iatrogenic injury following perianal surgery, particularly fistula repair. The risk of postoperative incontinence after fistula surgery ranges from 11% to 20%, depending on fistula type, location, and surgical technique [12]. The likelihood of incontinence increases proportionally with the amount of sphincter muscle divided during surgery.

Patient comorbidities, including diabetes mellitus [13], neurological disorders [14], a history of perianal or colorectal surgery [15], and obstetric factors [16], should be carefully evaluated. Digital rectal examination remains essential before further diagnostic investigations.

Clinical scoring systems are widely used to assess the frequency and severity of FI. The most common are the Cleveland Clinic Fecal Incontinence Score (Wexner score) [17], the fecal incontinence severity index (FISI) [18], and the fecal incontinence quality of life (FIQOL) scale [19]. The Wexner score ranges from 0 to 20, with higher scores indicating greater severity; a score of 8 or below generally indicates adequate continence [17].

Several therapeutic modalities are available for FI, although none provides complete success. The treatment approach depends on the severity and etiology of the condition [4]. Initial management includes conservative measures such as dietary modification, fiber supplementation, and bowel habit training [20, 21].

Biofeedback therapy is the first-line treatment for patients who do not respond to conservative measures [1]. Randomized controlled trials have reported long-term success rates of up to 86% [22, 23]. Therefore, biofeedback therapy is strongly recommended by professional societies, including the American College of Gastroenterology and the Rome Foundation [4, 21, 24]. However, conventional biofeedback has several limitations, including limited accessibility, the requirement for trained personnel, multiple clinic visits, patient embarrassment, and high cost.

The aim of this study was to evaluate the efficacy of home-based biofeedback therapy in patients with fecal incontinence and to identify factors associated with treatment success.

## Materials and methods

### Study design

This was a prospective, single-center pilot study conducted at Marmara University Hospital. The study protocol was approved by the local institutional ethics committee.

### Eligibility criteria

Patients presenting with symptoms of fecal incontinence were evaluated for eligibility. Inclusion criteria were age over 16 years and the absence of complete anal sphincter damage on neurological examination. Exclusion criteria included the presence of perianal fistula, rectal prolapse, congenital neurological disorders, severe dementia, severe psychiatric disease, genetic muscular disorders, or spinal trauma. Written informed consent was obtained from all patients who agreed to participate.

### Procedure and data collection

Clinical data of patients including age, sex, comorbidities, etiology of incontinence, history of radiotherapy, duration of symptoms, and type of incontinence were collected prospectively from digital hospital records. All patients underwent a detailed anal examination to assess anal tone, and an anal incontinence follow-up form was completed. Patients were questioned regarding demographic data, comorbidities, obstetric history (including normal or assisted vaginal deliveries), prior rectal injury repairs, perianal diseases, and any colorectal surgeries for benign or malignant conditions.

Fecal incontinence severity was evaluated using the Wexner score [17] fecal incontinence severity index (FISI) [25], and fecal incontinence quality of life (FIQOL) scale [26]. Previous treatment methods for fecal incontinence were also recorded. Anal manometry, anal electromyography, perianal magnetic resonance imaging (MRI), or endoanal ultrasonography were performed when available.

### Biofeedback treatment

Biofeedback therapy was recommended as the first-line treatment for fecal incontinence. Conventional anal biofeedback is typically performed in outpatient settings by trained healthcare professionals (physicians or nurses). However, the high cost, need for multiple hospital visits, and patient embarrassment often limit its accessibility.

To overcome these limitations, patients were offered a home-based biofeedback treatment using a novel device developed by our clinic. This device was designed to be simple, low-cost, and easy to use independently at home. The home biofeedback device consisted of three main components:

- a sphygmomanometer (for pressure measurement),
- an anal biofeedback balloon catheter, and
- a rotatable visual biofeedback scale that allows patients to monitor pressure changes during exercises

**Image 1:**
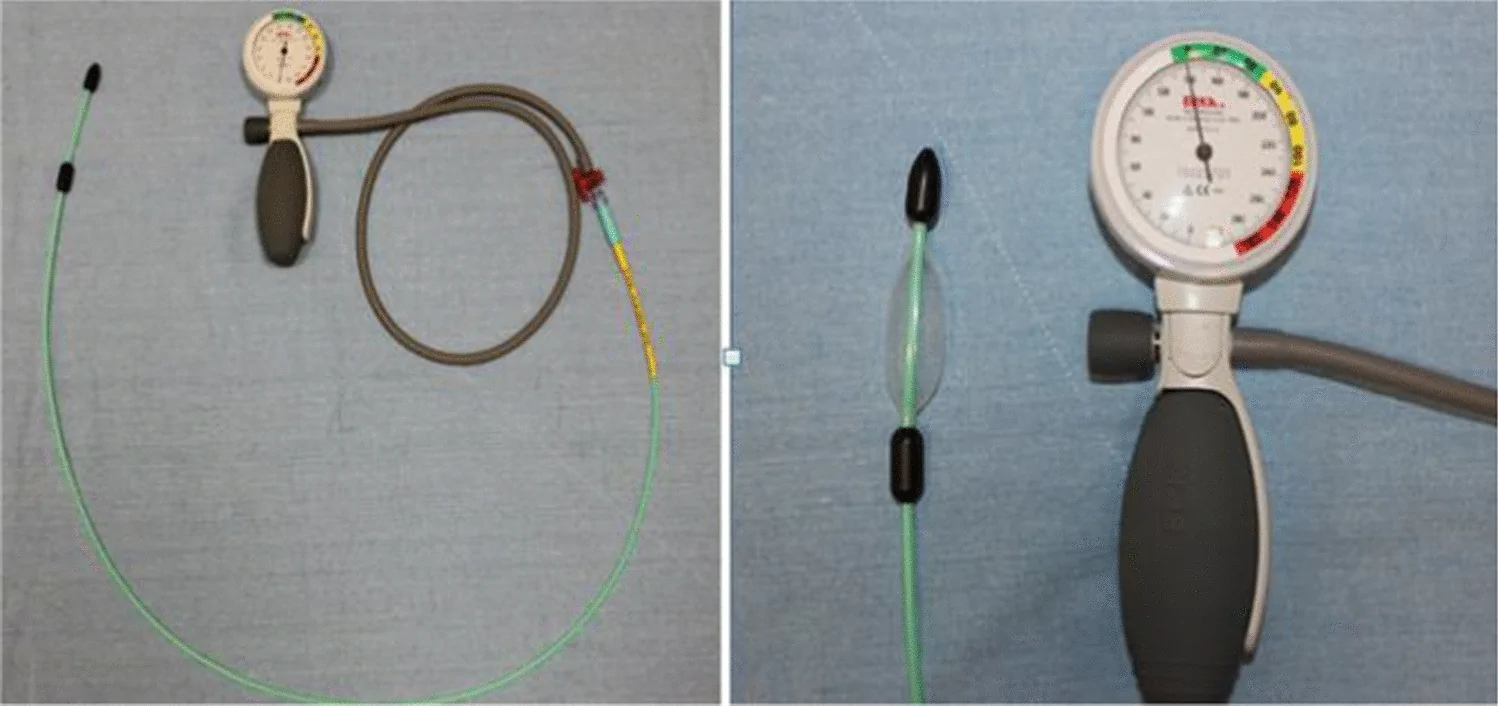
Home type anal biofeedback device

In the home-based biofeedback device we developed, the visual feedback pressure dial was categorized into three color-coded zones to facilitate ease of use and self-monitoring across all age groups: 0–60 mmHg (red), 60–120 mmHg (yellow), and 120–160 mmHg (green). This design enables patients to readily track their progress without the need for additional recording tools and allows for a standardized terminology when discussing outcomes with their physician.

### Biofeedback exercises

In this study, after inserting the inflated balloon catheter into the anal canal, the patient calibrates the device at rest by aligning the zero point of the ring with the current position of the needle. The patient is then instructed to perform anal sphincter contractions.

The primary purposes of the calibration ring is to establish a baseline prior to exercise and to provide visual feedback during training. This visual feedback is designed in a simple manner, encouraging progression from the red zone toward the green zone. At the same time, patients can follow the increasing of the squeezing pressure through in the calibration ring which is at the same time is visual feedback scale. The patients are guided to increase the pressure, with a target of 100 mmHg, thereby promoting conscious, regular strengthening of the external anal sphincter.

Importantly, contraction of accessory muscle groups (thigh and gluteal muscles) does not produce a meaningful change in the visual pressure dial. This distinction is clearly demonstrated to patients during the initial training session, and they are specifically instructed to avoid engaging these muscles. Owing to this mechanism, the home-based biofeedback device functions as a targeted physiotherapy focusing on the external anal sphincter, which is the primary structure responsible for anal incontinence.

After initiation of home-based biofeedback therapy, patients were scheduled for a follow-up visit at 1 week to assess whether the exercises were being performed correctly. In cases of improper application, patients received additional training and clarification regarding any misunderstood aspects. Subsequently, patients were instructed to continue the therapy at home by performing external anal sphincter contraction exercises twice daily (morning and evening) for 30 min per session, incorporating intermittent rest periods, over a period of 3 months.

Follow-up visits were scheduled at the first, second, and third months. At each visit, Wexner, FISI, and FIQOL scores were re-evaluated. The clinical efficacy of treatment was determined by comparing baseline and post-treatment scores.

**Image 2:**
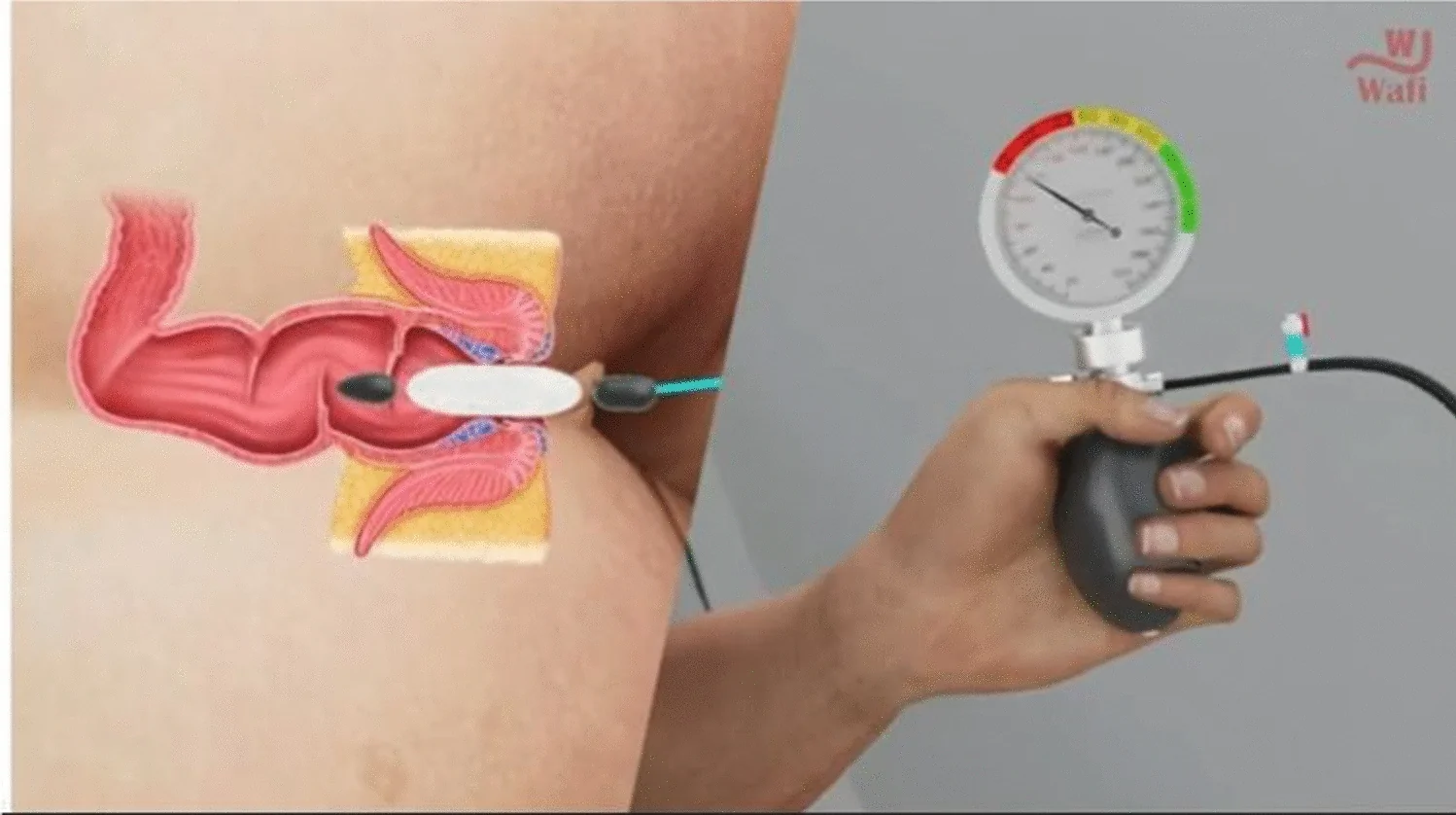
Illustration of home biofeedback training

### Statistical analysis

All statistical analyses were performed using SPSS software, version 23.0 (IBM Corp., Armonk, NY, USA). Data normality was assessed using the Shapiro–Wilk test. Continuous variables were expressed as mean ± standard deviation for normally distributed data, or as median (range) for non-normally distributed data. Comparisons between two independent groups were performed using the independent samples *t*-test for normally distributed data and the Mann–Whitney *U* test for nonparametric data. Paired comparisons of pre- and post-treatment variables within the same group were analyzed using the paired *t*-test or the Wilcoxon signed-rank test, as appropriate. Categorical variables were presented as frequencies and percentages, and comparisons between groups were made using the Chi-square or Fisher’s exact test when expected frequencies were small. Correlations between continuous variables were analyzed using Spearman’s rank correlation coefficient. All statistical tests were two-tailed, and a *p*-value < 0.05 was considered statistically significant.

## Outcomes

The primary outcomes were changes in Wexner, FISI, and FIQOL scores following home-based biofeedback treatment. The secondary outcomes were factors associated with treatment response.

## Results

### Patient selection and study flow

**Fig. 1:**
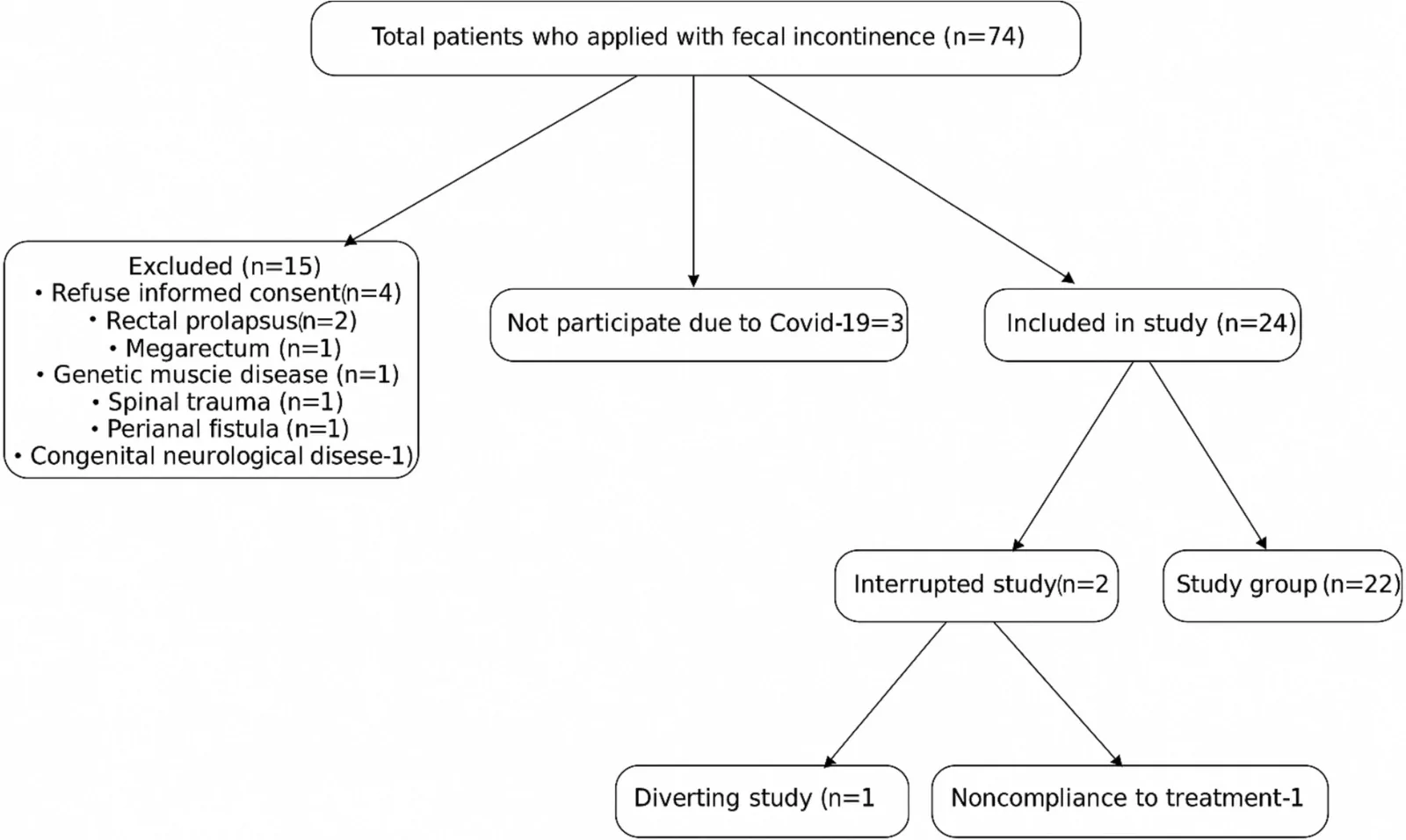
Flow chart showing patient selection, exclusion criteria, and final study group. A total of 74 patients with fecal incontinence were evaluated. A total of 15 patients were excluded for various reasons, and 35 patients did not participate owing to the COVID-19 pandemic. A total of 24 patients were included in the study; among them, two discontinued the study (one underwent diverting colostomy and one owing to noncompliance with treatment), resulting in a final study group of 22 patients

Patient characteristics and the inclusion process are summarized in Fig. 1. Between September 2019 and January 2021, a total of 74 patients who presented to the outpatient clinic with complaints of fecal incontinence were evaluated for eligibility. A total of 50 patients declined to participate in the study. A total of 35 patients refused to attend repeated hospital visits owing to the coronavirus disease 2019 (COVID-19) pandemic. Four patients refused to provide informed consent. One patient had a perianal fistula, two patients had grade 3 rectal prolapse for which surgical treatment was recommended, two patients had severe neurological impairment, three patients had severe dementia, one patient had schizophrenia, one patient had megarectum, one patient had a hypertensive anal sphincter, and one patient had a genetic muscular disease.

A total of 24 patients who met the inclusion criteria were enrolled in the study. Two patients discontinued participation: one owing to noncompliance with treatment and one owing to anastomotic stenosis requiring diverting ileostomy. The study was completed with 22 patients, and statistical analyses were performed on this cohort (Fig. 1).

### Patient characteristics

In the study group, the median age was 57 years (range, 17–69 years). A total of 14 patients (64%) were male and 8 (36%) were female. Ten patients (46%) had urinary incontinence in addition to fecal incontinence. The etiological cause was low anterior resection syndrome (LARS) in ten patients (46%). A total of 14 patients (64%) had fecal incontinence symptoms for more than 12 months (Table 1).

**Table 1:** Demographic and clinical characteristics of patients included in the study

Variable	*n* (%)/median (range)
Age (years)	57 (17–69)
Sex	Male 14 (64%) Female 8 (36%)
Comorbidities	Diabetes mellitus 4 (18%) Hypertension 6 (27%) Cerebrovascular disease 1 (5%) Hypothyroidism 2 (9%) Urinary incontinence 10 (46%)
Etiology	Perianal surgery 4 (18%) Obstetric trauma 4 (18%) Low anterior resection syndrome 10 (46%) Mild neurological damage 2 (9%) Idiopathic 2 (9%)
Prior radiotherapy	6 (27%)
Complaint period for fecal incontinence (months)	24 (1–360)
Incontinence subtype	Passive 8 (36.4%)

Data are presented as median (range) or number (%)

### Diagnostic evaluation and treatment outcomes

Of the patients included in the study, 21 (95%) had a Wexner score of 12 or higher. Anal manometry was performed in 17 patients (77%) before treatment; however, the procedure could not be carried out in 5 patients owing to the unavailability of a manometry catheter.

Endoanal ultrasonography (ERUS) was performed in seven patients (32%) prior to treatment. Sphincter muscle defects were detected in five of these patients (71%). Since ERUS could not be performed in the remaining patients, perianal magnetic resonance imaging (MRI) was used to evaluate anal sphincter anatomy. Perianal MRI was performed in eight patients (36%). When ERUS and MRI findings were assessed together, sphincter defects were identified in eight patients (36%) in total. Seven patients did not undergo either ERUS or perianal imaging. Anal electromyography (EMG) was performed in 21 patients (95%) before treatment, and partial axonal denervation was detected in 9 of them (43%) (Table 2).

**Table 2:** Evaluation according to pretreatment diagnostic tests

Variable	*n* (%)/median (range)
Wexner score (median)	18 (4–20)
Maximum squeeze pressure (median)	20 (0–100)
Endorectal ultrasound (ERUS)	7 (32%)
Anal electromyography (EMG)	21 (95%)
Perianal MRI	8 (36%)
Fecal incontinence treatment history	Lifestyle change/Diet: 22 (100%) Medical or surgery: 0 (0%)

Data are presented as median (range) or number (%)

The median duration of biofeedback treatment was 60 days (range, 7–90 days). Before treatment, the patients had high Wexner scores [median 18 (range, 4–20)], which decreased significantly after biofeedback therapy [median 6 (range, 0–16); *p* < 0.001]. The median fecal incontinence severity index (FISI) score was 48 (range, 20–57) before treatment and decreased to 15 (range, 0–45) after treatment, representing a statistically significant improvement (*p* < 0.001).

### Functional and quality of life outcomes

In manometric measurements, the median maximum squeeze pressure of the patients was markedly low before treatment [20 mmHg (range, 0–80 mmHg)], whereas a significant increase was observed after treatment [60 mmHg (range, 17–96 mmHg); *p* < 0.001].

According to the fecal incontinence quality of life (FIQL) scale, significant improvements were observed in all four major domains—lifestyle, coping/behavior, depression/self-perception, and embarrassment—after biofeedback therapy compared with baseline (*p* < 0.001) (Table 3).

**Table 3:** Fecal incontinence quality of life scale comparison

Parameter (median)	Before treatment	After treatment	*p* value
Lifestyle	12 (10–31)	38 (13–40)	< 0.001
Coping/behaviour	15 (8–29)	25 (13–36)	< 0.001
Depression/sense of self	12 (6–20)	24 (13–29)	< 0.001
Shame	5 (3–12)	11 (4–12)	< 0.001
Total	45 (31–83)	94 (43–117)	< 0.001

Data are presented as median (range)

When the maximum possible FIQL score (119) was considered, no patient achieved this score before treatment, whereas 11 patients (50%) reached a total FIQL score above 90 after treatment. Before treatment, 21 patients (95%) had severe incontinence (Wexner score > 8), while this rate decreased to 7 patients (40%) after treatment. Only three patients (14%) showed no change in their Wexner scores following biofeedback therapy.

Before treatment, ten patients (46%) had concomitant urinary incontinence in addition to fecal incontinence. Interestingly, following biofeedback therapy, five of these patients (50%) reported a significant improvement in urinary incontinence symptoms.

### Treatment response analysis

The remaining 19 patients (86%) demonstrated an improvement in Wexner scores ranging from 11% to 100% after treatment, indicating that while some patients experienced partial recovery, others achieved complete continence. In ten of these patients (53%), the improvement exceeded 50%. (Table 4).

**Table 4:** Comparison of groups according to treatment results

Parameter	Well response (*n* = 12)	Low response (*n* = 10)	*p* value
Age (median)	60 (17–69)	54 (24–69)	0.2
Sex	Male 7 (58%) Female 5 (42%)	Male 7 (70%) Female 3 (30%)	0.7
Diabetes mellitus	2 (17%)	2 (20%)	1.0
Low anterior resection syndrome (LAR)	5 (42%)	5 (50%)	0.7
Radiotherapy history	2 (17%)	4 (40%)	0.4
Anal surgery history	2 (17%)	2 (20%)	1.0
LARS	5 (42%)	5 (20%)	1.0
Neurological causes	1 (8%)	1 (10%)	1.0
Idiopathic	2 (17%)	0 (0%)	0.5
Incontinence time (median, months)	30 (1–360)	24 (1–156)	0.6
Treatment time (median, days)	68 (30–90)	60 (20–90)	0.3
Presence of pudendal neuropathy	5 (42%)	4 (40%)	1.0
Sphincter defect	3 (25%)	5 (50%)	0.4

Data are presented as median (range) or number (%)

To identify factors that might influence treatment outcomes, patients were divided into two groups: those who responded well to therapy (post-treatment Wexner score < 8) and those who responded poorly (post-treatment Wexner score > 8). A total of 12 patients (55%) were classified as good responders. No significant associations were found between treatment response and patients’ demographic characteristics, etiology of incontinence, duration of symptoms, treatment duration, or the presence of neuropathy or sphincter defects (Table 4).

## Discussion

In the present prospective study, we evaluated the effect of home-based biofeedback therapy in 22 patients with fecal incontinence treated at a single tertiary center. The results demonstrated a significant reduction in both median Wexner and fecal incontinence severity index (FISI) scores, as well as a marked improvement in patients’ quality of life following treatment.

Home-based biofeedback therapy was found to be effective in 86% of patients, with the degree of improvement ranging from 11% to 100%. More than half of the patients (53%) showed an improvement greater than 50% in Wexner scores.

Biofeedback therapy is considered the first-line treatment in patients with fecal incontinence who do not respond adequately to medical or conservative therapy. In our study, all patients were referred for biofeedback therapy and were followed prospectively to evaluate treatment efficacy.

The major strengths of the present study include its prospective design, the use of objective outcome measures, and the evaluation of a home-based biofeedback model, which reduces treatment costs and allows patients to perform the exercises independently and more frequently in their own environment. Moreover, the study contributes to the limited body of literature investigating the efficacy of anal biofeedback therapy.

However, our study also has limitations. The sample size was relatively small, the follow-up period was short, and not all patients could complete all diagnostic examinations. In addition, the COVID-19 pandemic affected patient follow-up and participation rates, which may have influenced the overall results.

In previous studies, there has been no standardized definition of symptomatic improvement or treatment success following biofeedback therapy for fecal incontinence. Liang et al. [27] conducted a prospective study involving 61 patients with anterior resection syndrome and defined an improvement of  > 15% in Wexner score as treatment success. After 15 sessions of office-based biofeedback therapy over 3 months, they reported a 66% success rate. Using the same threshold, the effectiveness rate in our study was calculated as 77%. The relatively high response rate in our study may be attributed to the home-based setting, which provided greater comfort and flexibility, allowing patients to perform exercises more regularly.

Similarly, Murad-Regadas et al. [28] evaluated 124 female patients treated with office-based biofeedback sessions twice a week (6–10 sessions) and defined clinical success as a > 50% improvement in Wexner scores after 6 months, with a 56% response rate. The rate observed in our study (53%) is comparable. Furthermore, we observed that improvement in Wexner scores was maintained during follow-up, consistent with the findings of Murad-Regadas et al.

Previous studies have not clearly defined which patient groups benefit most from biofeedback therapy. Liang et al.[27] reported better outcomes in patients who underwent laparoscopic anterior resection with pelvic nerve-sparing dissection. In our study, nine of ten patients with low anterior resection syndrome (LARS) had undergone open surgery, yet 70% of these patients achieved treatment effectiveness comparable to that described by Liang et al.

The potential effect of radiotherapy (RT) on treatment response remains controversial. Liang et al. [27] found that RT negatively influenced outcomes, and Allgayer et al. [29] reported poorer short-term responses in patients with a history of RT. By contrast, in our study, although six patients (27%) had a history of RT, prior RT did not significantly affect treatment outcomes. The small sample size may have contributed to this finding.

Leite et al. [30] demonstrated that the etiology of incontinence did not affect treatment response, a result consistent with our findings. Similarly, Murad et al. [28] found that a baseline Wexner score > 10 was associated with a poorer clinical response, whereas in our study, despite 95% of patients having a Wexner score > 10, most achieved satisfactory improvement. Gender was also not associated with treatment response in our series, consistent with the findings of Leite et al. [30].

No optimal duration for biofeedback therapy has been established in the literature. Mazor et al. [31] compared short-term (three sessions, 180 min) and long-term (six sessions, 315 min) office-based treatments and found no significant difference in clinical response or quality of life improvement. Similarly, in our study, no difference was observed between patients treated for less than 2 months and those treated for more than 2 months. The home-based protocol allowed patients to perform longer or more frequent sessions without logistical constraints, addressing one of the main limitations of traditional biofeedback therapy.

To date, there are no published data regarding the effect of anal biofeedback therapy on urinary incontinence accompanying fecal incontinence. Interestingly, in our study, half of the patients who had concomitant urinary incontinence showed symptomatic improvement after biofeedback therapy. We hypothesize that this improvement may result from reactivation and strengthening of the pelvic floor neuromuscular pathways involved in the continence mechanism. Future studies incorporating pre- and post-treatment urinary symptom questionnaires and objective voiding assessments are warranted to confirm this finding.

## Conclusions

Home-based biofeedback therapy appears to be an effective treatment option for patients with fecal incontinence. Owing to its simplicity, user-friendly design, and cost-effectiveness, it may be considered a practical alternative to conventional office-based biofeedback in appropriate settings. Nevertheless, further randomized controlled trials with larger patient populations and long-term follow-up are warranted to more comprehensively evaluate its efficacy, particularly in comparison with supervised outpatient rehabilitation and hybrid treatment protocols.

## Data Availability

The data that support the findings of this study are available from the corresponding author upon reasonable request.

## Declarations

### Generative AI and AI-assisted technologies in the writing process

The manuscript was originally prepared in Turkish and subsequently translated into English using the DeepL Translator (deepl.com/en/translator). To improve linguistic accuracy, readability, grammar, and overall language quality. AI-assisted tools, including ChatGPT (chat.openai.com), were used solely for editorial support. These technologies were applied exclusively for copy-editing purposes and did not contribute to the generation of original scientific content. Following the use of these tools, the author carefully reviewed and revised the manuscript and accepts full responsibility for the integrity and content of the final published version.
